# Does pre-enrichment of anodes with acetate to select for *Geobacter* spp. enhance performance of microbial fuel cells when switched to more complex substrates?

**DOI:** 10.3389/fmicb.2023.1199286

**Published:** 2023-11-23

**Authors:** Beate Christgen, Martin Spurr, Edward M. Milner, Paniz Izadi, Clare McCann, Eileen Yu, Tom Curtis, Keith Scott, Ian M. Head

**Affiliations:** ^1^School of Natural and Environmental Sciences, Newcastle University, Newcastle upon Tyne, United Kingdom; ^2^Environment Agency, Newcastle upon Tyne, United Kingdom; ^3^School of Engineering, Newcastle University, Newcastle upon Tyne, United Kingdom; ^4^Department of Environmental Microbiology, Helmholtz-Centre for Environmental Research—UFZ, Leipzig, Germany; ^5^Faculty of Health and Life Sciences, Northumbria University, Newcastle upon Tyne, United Kingdom; ^6^Department of Chemical Engineering, Loughborough University, Loughborough, United Kingdom

**Keywords:** microbial fuel cell, *Geobacter* spp., enrichment, acetate, wastewater

## Abstract

Many factors affect the performance of microbial fuel cells (MFCs). Considerable attention has been given to the impact of cell configuration and materials on MFC performance. Much less work has been done on the impact of the anode microbiota, particularly in the context of using complex substrates as fuel. One strategy to improve MFC performance on complex substrates such as wastewater, is to pre-enrich the anode with known, efficient electrogens, such as *Geobacter* spp. The implication of this strategy is that the electrogens are the limiting factor in MFCs fed complex substrates and the organisms feeding the electrogens through hydrolysis and fermentation are not limiting. We conducted a systematic test of this strategy and the assumptions associated with it. Microbial fuel cells were enriched using three different substrates (acetate, synthetic wastewater and real domestic wastewater) and three different inocula (Activated Sludge, Tyne River sediment, effluent from an MFC). Reactors were either enriched on complex substrates from the start or were initially fed acetate to enrich for *Geobacter* spp. before switching to synthetic or real wastewater. Pre-enrichment on acetate increased the relative abundance of *Geobacter* spp. in MFCs that were switched to complex substrates compared to MFCs that had been fed the complex substrates from the beginning of the experiment (wastewater-fed MFCs - 21.9 ± 1.7% *Geobacter* spp.; acetate-enriched MFCs, fed wastewater - 34.9 ± 6.7% *Geobacter* spp.; Synthetic wastewater fed MFCs – 42.5 ± 3.7% *Geobacter* spp.; acetate-enriched synthetic wastewater-fed MFCs - 47.3 ± 3.9% *Geobacter* spp.). However, acetate pre-enrichment did not translate into significant improvements in cell voltage, maximum current density, maximum power density or substrate removal efficiency. Nevertheless, coulombic efficiency (CE) was higher in MFCs pre-enriched on acetate when complex substrates were fed following acetate enrichment (wastewater-fed MFCs – CE = 22.0 ± 6.2%; acetate-enriched MFCs, fed wastewater – CE =58.5 ± 3.5%; Synthetic wastewater fed MFCs – CE = 22.0 ± 3.2%; acetate-enriched synthetic wastewater-fed MFCs – 28.7 ± 4.2%.) The relative abundance of *Geobacter* ssp. and CE represents the average of the nine replicate reactors inoculated with three different inocula for each substrate. Efforts to improve the performance of anodic microbial communities in MFCs utilizing complex organic substrates should therefore focus on enhancing the activity of organisms driving hydrolysis and fermentation rather the terminal-oxidizing electrogens.

## Introduction

Microbial fuel cells (MFCs) are bioelectrochemical reactors which can produce electricity from organic substrates. These can be simple substrates such as acetate or complex mixtures of organic compounds such as wastewater. In its simplest form, a bioelectrochemical system (BES) consists of an anode, a cathode and a microbial catalyst which can be associated with the anode, the cathode or both (Logan et al., 2006; Rinaldi et al., 2008; Christgen et al., 2020). Exoelectrogenic bacteria have the ability to transfer electrons, from oxidation of organic or inorganic compounds out of the cell to other organisms (e.g., methanogens), an insoluble electron acceptor such as iron oxides or to an electrode in MFCs (Sydow et al., 2014; Logan et al., 2019; Bird et al., 2021; Thapa et al., 2022). In the case of an MFC the electrons are transferred through an external circuit to the cathode where they are used to reduce an electron acceptor (e.g., oxygen) and generate an electrical current (Logan, 2009; Logan and Rabaey, 2012; Prokhorova et al., 2017).

The substrate, as the electron donor and thus fuel for the MFC is a critical factor for the development of the anodic community and is one of the principal biological factors affecting electricity generation (Liu et al., 2009; Pant et al., 2010). A wide variety of substrates has been tested in MFCs to generate electricity (Chae et al., 2009; Pant et al., 2010; Pandey et al., 2016; Mateo et al., 2018; Prathiba et al., 2021). Acetate has been used extensively as an electron donor in MFCs and frequently leads to the selective enrichment of electroactive bacteria in the anode community especially *Geobacter* spp. (Liu et al., 2005; Logan and Rabaey, 2012; Zhu et al., 2014b; Kumar et al., 2015; Eyiuche et al., 2017).

Acetate is a central product from the hydrolysis and fermentation of complex carbon sources and is widely used as an electron donor and carbon source for terminal oxidation processes such as methanogenesis, iron-reduction or sulfate reduction, as well as electron transfer to an anode in MFCs. High coulombic efficiency is often observed in MFCs fed acetate which is sometimes considered as a benchmark for MFC performance (Mcinerney et al., 2009; Pant et al., 2010). However, in application of MFCs for recovery of energy from wastewater for example, more complex substrates are the norm with acetate accumulating at low levels as an intermediate in anaerobic microbial food chains. MFCs fed complex substrates typically show lower performance and higher microbial diversity on the anode than those fed acetate (Zhang et al., 2011; Heidrich et al., 2018). One explanation for this could be that acetate produced by fermentation is consumed by diverse non-electrogenic bacteria as suggested by Chae et al. (2009). Thus insufficient electrogens are present to effectively generate electricity from acetate generated from hydrolysis and fermentation of complex substrates present in wastewater (Chae et al., 2009). We have tested the hypothesis, that pre-enrichment of anodic biofilms with acetate improves MFC performance owing to enrichment of exoelectrogens at the anode which in turn reduces the amount of acetate consumed by organisms that do not contribute to energy generation. This was done by switching acetate fed MFC to synthetic wastewater or real domestic wastewater and comparing their performance and anodic microbial communities to MFC that were fed either synthetic wastewater (OECD, 2010) or real domestic wastewater from the start. We also tested whether the inoculum used (MFC effluent, activated sludge and Tyne River sediment) had any impact on MFC performance and the enriched anodic communities.

## Materials and methods

### Single chamber MFC configuration and operation

Forty-five MFC reactors were prepared. All of the MFC were enriched and operated under closed circuit conditions with an external resistance of 100 ohms. The MFCs were inoculated with either (i) MFC effluent (MFC) from previously set up acetate-fed MFC reactors; (ii) Tyne River sediment (TS); (iii) activated sludge (AS). Replicate MFCs for each inoculum were enriched with either (i) acetate, (ii) synthetic wastewater (OECD medium), (iii) municipal wastewater (WW), (iv) acetate followed by synthetic wastewater (Ac-OECD) or (v) acetate followed by municipal wastewater (Ac-WW) as substrates. Each treatment was prepared in triplicate (3 replicates x 3 inocula x 5 enrichment media). In addition, 15 control reactors were set up in the same way (1 reactor per 3 inocula x 5 enrichment media) and operated under open circuit conditions.

Each MFC reactor had a working volume of 200 mL. The anode material was carbon veil (Technical Fiber Products LTD, United Kingdom) and the air cathode comprised a membrane electrode assembly made from an anion exchange membrane (Fumasep FAD-PET-75, Fumatech BWT GmbH, Germany), a gas diffusion electrode (GDE) consisting of carbon paper (Freudenberg, Germany) with a catalyst load of 0.8 mg Pt/cm^2^ using nafion as binder and a titanium sheet as a current collector. The anode was placed in a polyethylene terephthalate (PET) frame connected to a datalogger using titanium wire. The cathode area was 12.5 cm^2^ which was three times the area of the anode (4.2 cm^2^) to ensure the cathode was not limiting. Anode and cathode were 3 cm apart. A 100 Ω resistor was included in the circuit and an Ag/AgCl reference electrode (BASi, United States) connected using a salt bridge (3 M KCl in agar as electrolyte, +0.197 V vs. SHE) which was positioned directly next to the anode was used to enable continuous monitoring of the anode potential with the cell voltage using a datalogger (DT85 data logger, Omni Instruments, United Kingdom).

Acetate medium comprised 540 mg/L sodium acetate, 5.02 g/L HK_2_PO_4_, 2.88 g/L H_2_KPO_4_, 280 mg/L NH_4_Cl, 100 mg/L MgSO_4_.7 H_2_O, 4.3 mg/L CaCl_2_, 4 mg/L FeCl_2_.4H_2_O, 2 mg/L CoCl_2_.6H_2_O, 2 mg/L NiCl_2_.6H_2_O, 1 mg/L MnCl_2_.4 H_2_O, 0.32 mg/L Na_2_SeO_3_, 0.14 mg/L (NH_4_)_6_Mo_7_O_24_.4H_2_O, 0.1 mg/L H_3_BO_3_, 0.1 mg/L ZnCl_2_, 0.08 mg/L CuCl_2_.2H_2_O, 0.004 mL/L 35% HCl, 1 mg/L pyridoxine hydrochloride, 0.5 mg/L nicotinic acid, 0.25 mg/L riboflavin, 0.25 mg/L thiamine hydrochloride, 0.2 mg/L biotin, 0.2 mg/L folic acid, 0.01 mg/L vitamin B_12_. Organization for Economic Cooperation and Development synthetic wastewater medium (OECD, comprised 160 mg/L peptone, 110 mg/L yeast extract, 30 mg/L urea, 28 mg/L K_2_HPO_4_, 3 g/L NaCl, 4 mg/L CaCl_2_.2H_2_O, 2 mg/L MgSO_4_.7H_2_O). Municipal wastewater was collected from Tudoe Mill sewage treatment works (Northumbrian Water, County Durham, United Kingdom). The chemical oxygen demand (COD) and conductivity of the three substrates was matched by adding salts (3 g/L NaCl) to match the highest conductivity (acetate medium with a conductivity of 5.46 mS at pH 7.04). The reactors were batch fed and considered enriched when the peak voltage was reproducible over three consecutive cycles at which point electrochemical (polarization, cyclic voltammetry, electrochemical impedance spectroscopy) and analytical (chemical oxygen demand; COD, pH, conductivity) measurements were made at the end of each batch cycle. Following this, the reactors were refilled. In total the reactors were operated for over 3 months.

The COD was measured according to the APHA standard method (Eaton et al., 1998). The conductivity of the substrate was measured using a handheld conductivity meter pIONeer 30 (Radiometer Analytical, France) and the pH was measured using a portable 3,310 pH Meter (Jenway, United Kingdom).

Once the voltage stabilized, the reactors were polarized. During polarization the change in cell current and voltage, as well as the anode potential (vs. an Ag/AgCl reference electrode), were recorded continuously using a data logger (DT85 data logger, Omni Instruments, United Kingdom). Polarization curves were recorded starting at open circuit potential (OCP) using a potentiostat (DropSense STAT800 multichannel potentiostat, Metrohm, United Kingdom) at a scan rate of 1 mV s^−1^. The internal resistance was measured by electrochemical impedance spectroscopy using a potentiostat (DropSense STAT800 multichannel potentiostat, Metrohm, United Kingdom). Impedance measurements were conducted at OCP over a frequency range of 30,000 to 0.1 Hz with a sinusoidal perturbation of 10 mV amplitude. The coulombic efficiency (CE) was calculated as detailed in Logan et al. (2006).

### DNA extraction and microbial community structure analysis

At the end of the experiment the anodes were removed from the reactors and stored in 50% ethanol for microbial analysis. For control reactors enriched and operated under open circuit conditions DNA was extracted from the non-polarized carbon veil electrodes. The entire anodes and non-polarized electrodes were homogenized mechanically using a sterile pestle (Merck, Germany) and vortexed for 3 min. Two ml of supernatant was removed and spun down at 14,000 rpm for 10 min to pellet the cells. The supernatant was removed. The pellet was suspended in 978 μL sodium phosphate buffer and added to the lysing tube from a FastDNA Spin Kit for Soil (MP Biomedicals, Germany). The DNA was extracted following the manufacturer’s instructions.

16S rRNA genes were amplified from total DNA and the V4 variable region was sequenced following the 16S Illumina Amplicon Protocol from the Earth Microbiome Project (Kozich et al., 2013) at NUOmics (Northumbria University, Newcastle upon Tyne). Raw sequence data (FastQ files) obtained from the Illumina sequencing platform were demultiplexed and analyzed using QIIME 2 (Caporaso et al., 2010). DADA2 was used for amplicon sequence variant (ASV) selection (Callahan et al., 2016). ASVs which appeared in the negative control were removed from the other samples. Phylogenetic trees were constructed using representative sequences of dominant taxonomic groups. BLASTN was used to identify nearest neighbors (Altschul et al., 1997). The tree is based on comparative analysis of the most abundant partial 16S rRNA sequences. The phylogeny was inferred using the Neighbor-Joining method (Saitou and Nei, 1987). The optimal tree with the sum of branch length = 1.71425693 is shown. The percentage of replicate trees in which the associated taxa clustered together in the bootstrap test (1,000 replicates) are shown next to the branches (Felsenstein, 1985). The tree is drawn to scale, with branch lengths in the same units as those of the evolutionary distances used to infer the phylogenetic tree. The evolutionary distances were computed using the Maximum Composite Likelihood method (Tamura et al., 2004) and are in the units of the number of base substitutions per site. This analysis involved 30 nucleotide sequences. All ambiguous positions were removed for each sequence pair (pairwise deletion option). There were a total of 227 positions in the final dataset. Evolutionary analyzes were conducted in MEGA X (Kumar et al., 2018).

### Statistical analysis

All statistical analyzes were conducted in PAST 4 (Hammer et al., 2001). Kruskal-Wallis was used to determine if significant differences were present and the significance of differences was determined using Tukey’s honestly significant difference (HSD). A value of p of less than 0.05 was considered to indicate statistical significance.

## Results

### Effect of substrate, inoculum and acetate pre-enrichment on MFC performance

**Figure 1:**
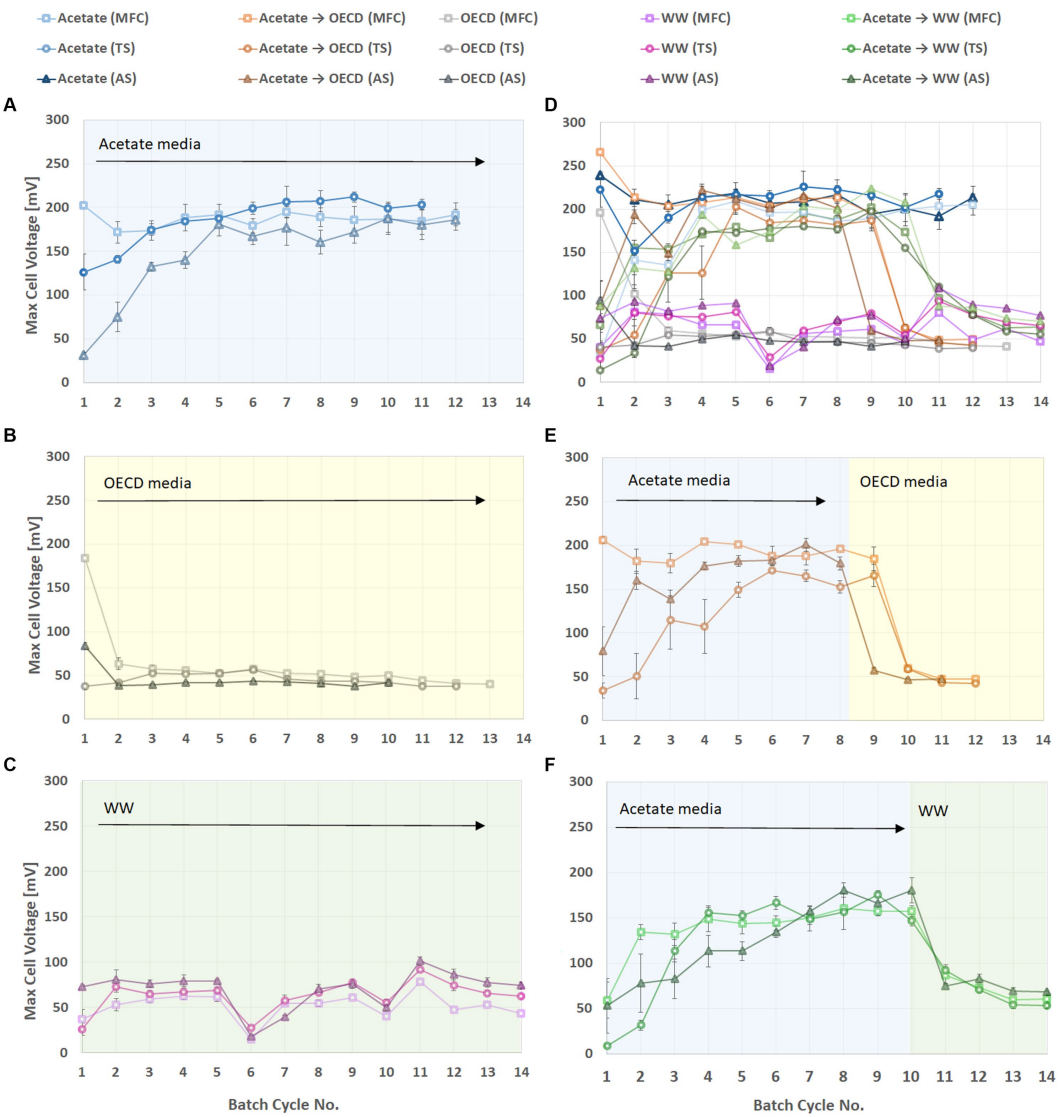
Cell voltage of triplicate MFC reactors inoculated with MFC effluent, Tyne sediment or activated sludge and fed different substrates. **(A)** Reactors fed acetate. **(B)** Reactors fed synthetic wastewater (OECD medium). **(C)** Reactors fed domestic wastewater. **(D)** Reactors enriched on acetate medium until stable performance was observed and switched to domestic wastewater. **(E)** Reactors enriched on acetate medium and switched to OECD medium. **(F)** Data from all reactors combined in one graph to illustrate overall performance trends.

After feeding with 3 to 4 batches of substrate all reactors were enriched and showed stable voltage generation (Figure 1). The voltage generated during a batch depended on the substrate fed to the MFC (Figure 1). For reactors enriched on acetate medium and then switched to OECD (Figure 1E) and WW (Figure 1D) an immediate voltage drop was was observed. The highest voltage was observed for reactors using acetate medium as substrate (on average 178.3 ± 26.1 mV). Reactors fed on the more complex substrates, OECD medium and WW, exhibited lower cell voltage. This was on average 45.9 ± 7 mV for reactors fed OECD medium and 61.7 ± 19.8 mV for wastewater fed reactors (Figure 1). Wastewater is the least defined and most variable substrate used, and this is clearly visible in the variability of the observed cell voltage (Figure 1C). The reactors enriched on acetate medium and then switched to OECD and WW exhibited a slight, but not statistically significant (*p* > 0.05), increase in cell voltage with values of on average 49.9 ± 7.2 mV and 66.8 ± 10.8 mV, respectively.

Small but statistically significant (*p* < 0.05) differences in cell voltage were observed for reactors set up with different inocula. Of the reactors fed with acetate medium, those inoculated with activated sludge (AS) generated an average cell voltage of 186 ± 24.6 mV, those inoculated with MFC effluent (MFC) had a lower voltage (177.9 ± 26.2 mV) and those with Tyne sediment (TS) inoculum had the lowest voltage (166 ± 24.1 mV). Wastewater-fed reactors showed a similar trend in relation to activated sludge as inoculum with the highest voltage generated of 69.8 ± 20.1 mV but reactors inoculated with TS generated higher voltage (64.1 ± 14.9 mV) than reactors inoculated with MFC effluent (53.9 ± 15.6 mV). Reactors using OECD medium as a substrate produced on average 49 ± 6.6 mV when inoculated with MFC effluent, 45.1 ± 6.3 mV when inoculated with TS and 44.1 ± 6.3 mV when inoculated with AS. It was also apparent that reactors inoculated with MFC effluent had the highest initial potential, which was maintained in acetate-fed reactors but decreased rapidly in OECD-fed reactors or started at a much lower level in WW-fed reactors (Figure 1).

**Figure 2:**
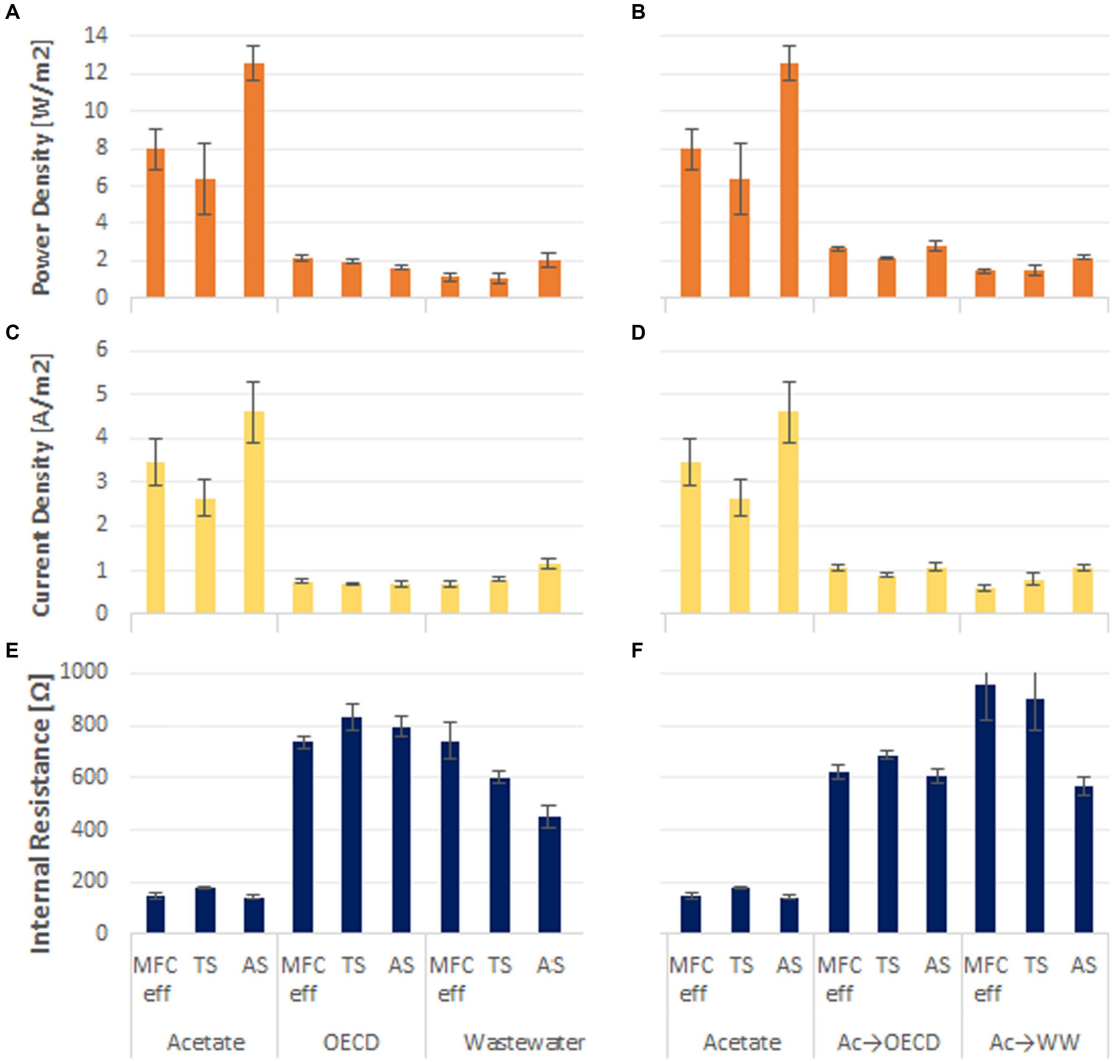
Power density of the triplicate MFC reactors fed **(A)** acetate, OECD medium and domestic wastewater, **(B)** acetate compared to reactors enriched on acetate and switched to OECD medium and WW; Current density of the MFC reactors fed **(C)** acetate, OECD medium and WW; **(D)** acetate compared to reactors enriched on acetate and switched to OECD medium and WW; Internal resistance of the MFC reactors fed **(E)** acetate, OECD medium and WW; **(F)** acetate compared to reactors enriched on acetate and switched to OECD medium and WW.

The power density that a reactor can achieve (Figures 2A,B) was determined from polarization curves. The highest power density and current density was obtained in acetate fed reactors (8.98 ± 1.33 W/m^2^ at 3.5 ± 0.8 A/m^2^). This was approximately four times greater than the power and current densities observed in reactors fed OECD medium and domestic wastewater (1.9 ± 0.12 W/m^2^ at 0.89 ± 0.2 A/m^2^ and 1.69 ± 0.13 W/m^2^ at 0.72 ± 0.03 A/m^2^ for OECD and WW respectively, Figures 2A,C). Acetate enriched reactors fed with OECD showed the second highest peak power and current densities with 2.52 ± 0.18 W/m^2^ at 1.02 ± 0.08 A/m^2^, while, acetate enriched reactors fed with WW achieved 1.69 ± 0.15 W/m^2^ at 0.84 ± 0.2 A/m^2^ (Figures 2B,D).

In acetate-fed reactors the inoculum used impacted the peak power density achieved. Reactors inoculated with activated sludge achieved 50% higher peak power than reactors inoculated with MFC effluent and Tyne sediment (Figure 2B).

The internal resistance of the reactors exhibited the opposite trend to peak power. Acetate-fed reactors had the lowest internal resistance (156.1 ± 8 Ω) with the OECD-fed reactors showing 5 times higher internal resistances (786 ± 37 Ω) and the wastewater-fed reactors 4 times higher internal resistance (596 ± 45 Ω). Acetate-enriched reactors fed OECD had 4 times higher internal resistance (638 ± 24 Ω), and Acetate-enriched reactors fed WW had 5 times higher internal resistance (808 ± 95 Ω) than purely acetate-fed MFC. Control reactors enriched and operated under open circuit conditions had high internal resistance (on average 32,978 ± 8,437 Ω) (Supplementary Figure S1), and all had the same open circuit potential of on average 811.8 ± 6.7 mV (Supplementary Figure S2).

### Effect of substrate, inoculum and acetate pre-enrichment on substrate removal performance and coulombic efficiency

**Figure 3:**
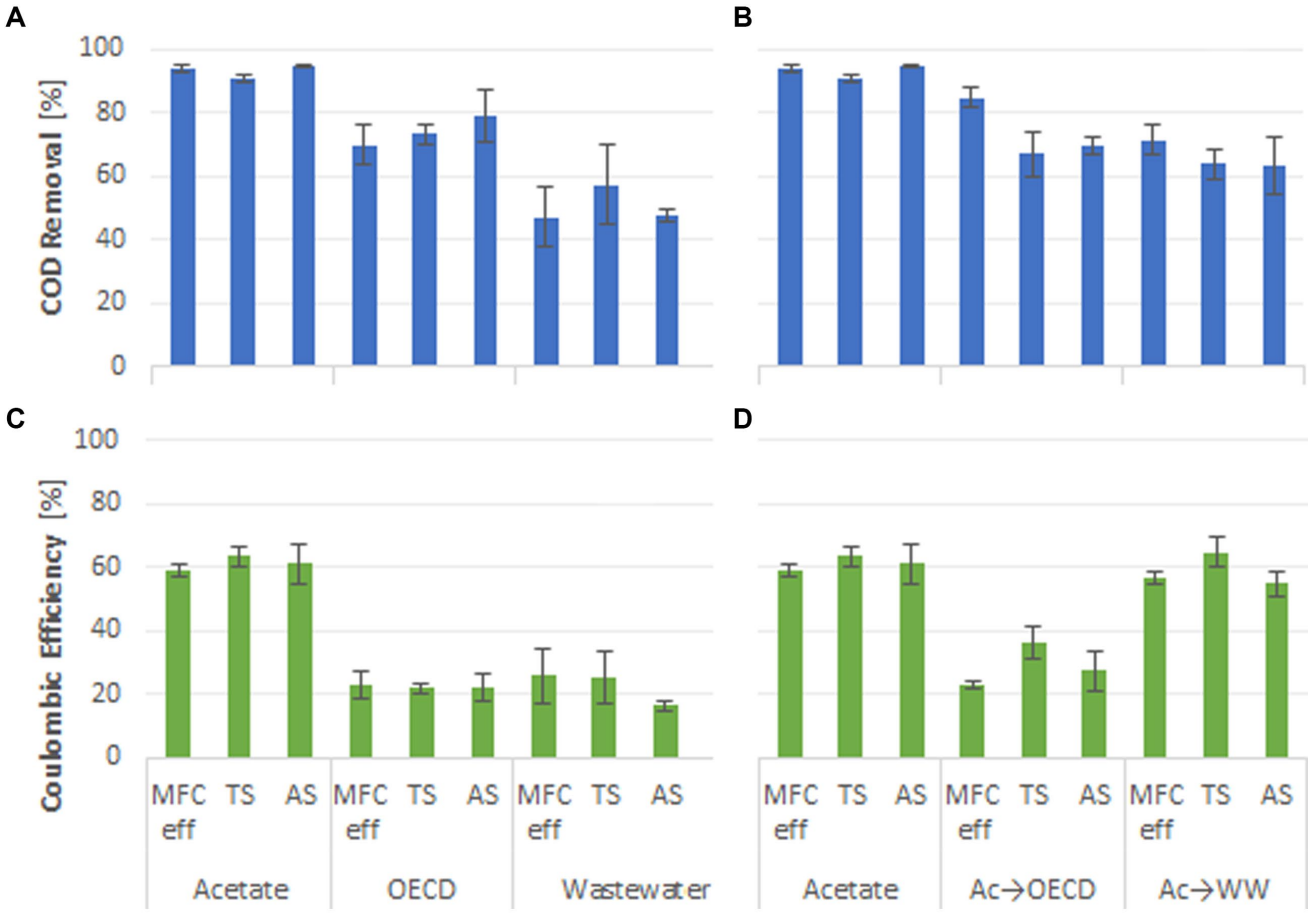
Percentage COD removal of the MFC reactors fed **(A)** acetate, OECD medium and WW; **(B)** acetate compared to reactors enriched on acetate and switched to OECD medium and WW; CE of the MFC reactors fed **(C)** acetate, OECD medium and WW; **(D)** acetate compared to reactors enriched on acetate and switched to OECD medium and WW.

The percentage COD removal was used as an indicator of substrate removal efficiency in the MFC reactors. Acetate-fed reactors had significantly higher substrate removal than OECD- and WW-fed reactors and the acetate-enriched reactors fed OECD and WW (Figure 3). The highest COD removal was observed in reactors fed acetate medium with 93 ± 0.8% COD removal (Figure 3). Reactors fed OECD medium, acetate-enriched reactors fed OECD medium, acetate-enriched reactors fed WW and reactors fed-WW alone had lower substrate removal efficiencies (74 ± 5.9%, 73 ± 4.2%, 66 ± 6.1% and 50 ± 7.9% COD removal respectively) (Figure 3). Control reactors enriched and operated under open circuit conditions exhibited high level of COD removal with 95.2 ± 1.8%, 90.7 ± 1.8%, 88.6 ± 4.7%, 76.9 ± 10.9%, and 68.2 ± 5.3% COD removed for control reactors fed on acetate, acetate-enriched reactors switched to OECD, OECD, acetate-enriched reactors switched to WW, and WW, respectively (Supplementary Figure S3).

Coulombic efficiency (CE) is a measure of the proportion of electrons produced from oxidation of the substrate that is used for electricity generation. Simple substrates, such as acetate, that are known to enrich for, and be oxidized by, exoelectrogens typically display high CEs (Pant et al., 2010). Consistent with this, on average 61 ± 3.9% of the electrons produced were recovered as electricity in acetate-fed reactors (Figure 3). However, acetate-enriched reactors fed WW as substrate also exhibited high CEs (58.5 ± 3.5%). The lowest CEs were observed for OECD- and WW-fed reactors with coulombic efficiencies of 22.0 ± 3.2% and 22.2 ± 6.2%. Acetate-enriched OECD-fed reactors had a 6 % higher CE of 28.7 ± 4.2%, compared to reactors solely fed OECD, without pre-enrichment on acetate. An even greater uplift in CE was observed in WW-fed MFC pre-enriched on acetate compared to reactors fed WW alone (58.5 ± 3.5% compared to 22.2 ± 6.2%). There was no significant difference for wastewater treatment performance or CE for reactors fed the same substrate with different inocula (*p* > 0.05; Figure 3). The current flow at OCP was essentially zero (Supplementary Figure S4) and consequently CE was also zero. The primary factor dictating COD removal and coulombic efficiency was the feed substrate with pre-enrichment on acetate having a significant positive effect on coulombic efficiency when MFC feed was switched to more complex substrates.

### Effect of substrate, inoculum and acetate pre-enrichment on anodic microbial community diversity and composition

Alpha diversity is the mean species diversity in a habitat at a local scale. The Shannon Index, which takes into account evenness and richness of a sample, is one measure of alpha diversity and shows that with increasing complexity of the substrate, the diversity of the anodic microbial community also increases. For example, in reactors fed acetate the Shannon Index was 1.9 ± 0.22 while in reactors enriched on acetate and then fed on OECD or WW the Shannon Index was 2.6 ± 0.013 and 3.5 ± 0.14, respectively (Supplementary Figure S5). Reactors inoculated with MFC inoculum presented a slightly lower alpha diversity for all reactors except those fed WW (Supplementary Figure S5). Higher alpha diversity was observed in the control reactors with TS and AS inoculum but not in reactors with MFC effluent as inoculum or the control reactors operated under open circuit potential (OCP) fed acetate with MFC effluent as inoculum (Supplementary Figure S5). The alpha diversity for the open circuit control reactors ranged from 1.43 (lowest Acetate fed MFC inoculum) to 3.94 (highest Wastewater fed Tyne sediment inoculum) (Supplementary Figure S5).

**Figure 4:**
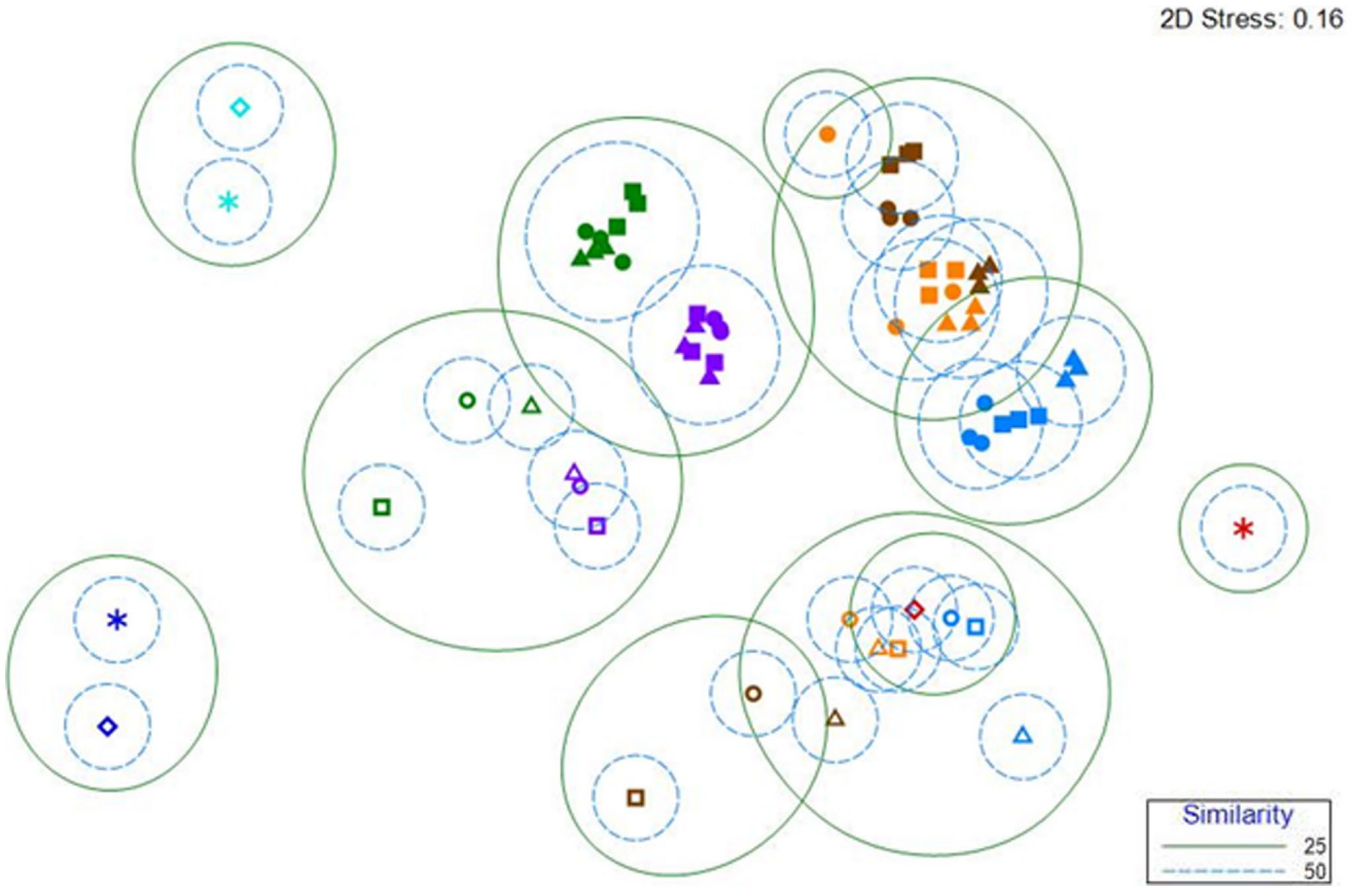
nMDS clustering of Bray–Curtis similarity of microbial community data for MFC, control reactors, and inocula. The analysis was conducted at the family level. The symbols are colored by treatment. Blue, acetate fed reactors; Orange, acetate-enriched, OECD-fed reactors; Brown, OECD fed reactors; Purple, acetate-enriched, WW-fed reactors; Green, WW-fed reactors. Closed symbols represent MFC, open symbols represent control reactors enriched and operated in open circuit. Triangles represent reactors inoculated with MFC effluent, Squares represent reactors inoculated with Tyne Sediment and Circles represent reactors inoculated with Activated Sludge. Stars and open diamonds represent samples of the inoculum (Turquoise-activated sludge inoculum; Red-MFC effluent inoculum; Dark Blue-Tyne Sediment inoculum).

Clustering the reactor, control and inoculum samples using Bray–Curtis similarity demonstrated that the primary factor dictating anodic microbial community composition is the substrate fed to the reactors (Figure 4). The communities from acetate-fed reactors (Figure 4, filled blue symbols) are distinct from those in acetate-enriched reactors switched to OECD samples (Figure 4, filled orange symbols), which cluster with the communities from reactors fed OECD alone (Figure 4, filled brown symbols). The communities from acetate-fed reactors switched to WW are distinct from those fed WW alone (Figure 4, filled purple and green symbols respectively). The AS and TS inoculum communities (Figure 4, turquoise and dark blue stars and open diamonds) are distinct from all other communities, while one of the MFC inoculum communities (Figure 4, red star) is distinct, the second MFC inoculum community (Figure 4, open red diamond) clustered with control reactor samples operated at open circuit, especially the control reactors fed acetate. This may be explained by the MFC inoculum being taken from 2 different MFC reactors.

The microbial communities in control reactors enriched and operated at open circuit (Figure 4, open symbols) were distinct from the MFC communities. The communities from acetate-fed control reactors (Figure 4, open blue symbols) clustered with, but were distinct from communities from acetate-enriched control reactors switched to OECD (Figure 4, open orange symbols), which were intermediate between the communities from acetate-fed reactors (Figure 4, open blue symbols) and communities from reactors fed OECD alone (Figure 4, open brown symbols).

The communities from WW-fed open circuit control reactors (Figure 4, open green symbols) and acetate-enriched open circuit control reactors switched to WW (Figure 4, open purple symbols), were distinct from the other open circuit control reactors and their respective MFC reactor communities (Figure 4).

The data clearly show enrichment of distinct communities in the MFC reactors and community composition varied depending on the substrate fed to the MFC.

**Figure 5:**
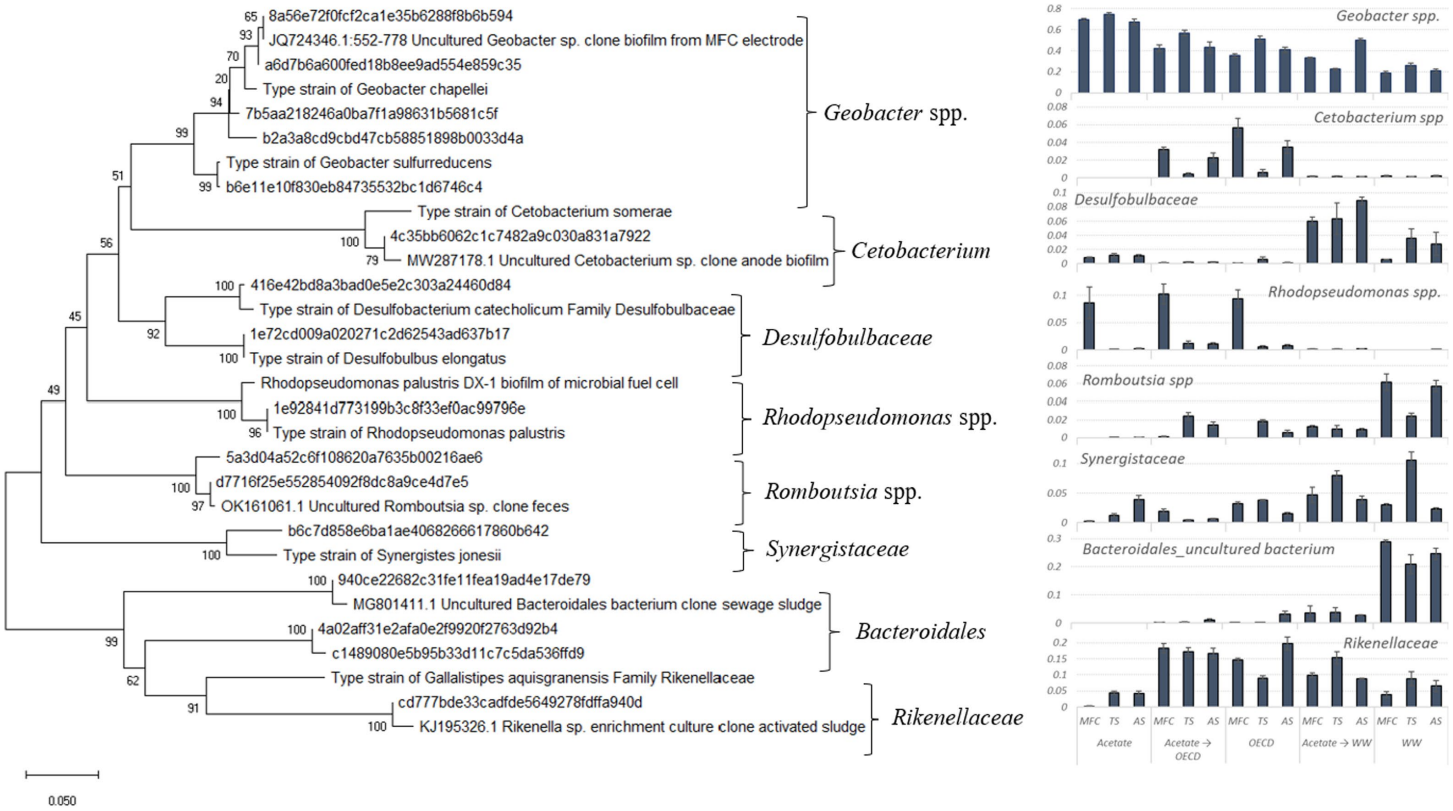
Phylogenetic distance tree of key bacterial taxa and close relatives (left) and plots of the mean fractional abundances of these taxa in MFC reactors (right). The tree is based on comparative analysis of the most abundant partial 16S rRNA sequences.

*Deltaproteobacteria* were enriched in all MFC reactors but particularly in acetate-fed reactors (Supplementary Figure S6). By contrast, in control reactors operated at open circuit and in the inocula, the phyla *Bacteroidia*, *Clostridia* and *Gammaproteobacteria* were most abundant. *Geobacter* was the single most abundant genus in the MFC reactors and was present at highest relative and absolute abundance in MFCs fed acetate (*p* < 0.05); 70.4 ± 1.7% relative abundance; equivalent to 1.1 ± 0.002 × 10^11^ *Geobacter* cells/cm^2^ of anode (Supplementary Figure S7). In acetate-enriched reactors fed OECD synthetic wastewater, *Geobacter* was present at a relative abundance of 47.3 ± 3.9%; equivalent to 2.2 ± 0.015 × 10^10^ *Geobacter* cells/cm^2^ of anode (Supplementary Figure S7), and in reactors fed OECD synthetic wastewater alone *Geobacter* was present at lower average relative and absolute abundance (42.5 ± 3.7%, equivalent to 1.2 ± 0.013 × 10^10^ *Geobacter* cells/cm^2^ of anode, Supplementary Figure S7), which with respect to both relative and absolute abundance was statistically significantly different from acetate-enriched OECD synthetic wastewater fed reactors (*p* < 0.05). MFCs fed WW had the lowest relative abundance of *Geobacter* spp. (*p* < 0.05) but acetate-enriched MFCs fed WW had a higher relative abundance of *Geobacter* spp. than MFCs fed WW alone (*p* < 0.05; 34.9 ± 6.7% and 21.9 ± 1.7% relative abundance respectively) (Figure 5). In control reactors enriched and operated at open circuit *Geobacter* spp. were present at a relative abundance of 7.03 ± 0.36; 0.66 ± 0.34; 0.31 ± 0.12; for reactors fed acetate, OECD medium and domestic wastewater, respectively. In control reactors enriched on acetate and switched to OECD medium and wastewater *Geobacter* spp. were present at a relative abundance of 2.9 ± 1.29 and 1.9 ± 0.74, respectively. Other taxa enriched in the MFC reactors present at greater than 1% relative abundance included the genus *Rhodopseudomonas* in MFC reactors inoculated with MFC effluent and fed acetate or OECD and in acetate-enriched OECD reactors (Figure 5). Members of the, genus *Cetobacterium* were also present at greater than 1% relative abundance in acetate-enriched MFC fed OECD and OECD fed reactors inoculated with MFC effluent and activated sludge. Members of the families *Rikenellaceae* and *Synergistaceae* were present in all MFC reactors at levels above 1% relative abundance and members of the genus *Romboutsia* were present in all MFC reactors fed complex substrates but were most highly enriched in reactors fed WW. A similar pattern was seen for bacteria from the order *Bacteroidales.* The family *Desulfobulbaceae* was most prominent in MFCs which have been operated with WW (Figure 5).

## Discussion

### Effect of substrate on electrical and wastewater treatment performance

The performance of MFC reactors in terms of maximum voltage, current density in batch operation and maximum power density achieved during polarization, demonstrated a clear trend of higher performance with decreasing substrate complexity (Figures 1–3). This is in line with results from (Velasquez-Orta et al., 2011) who observed a decrease in MFC performance with an increase in substrate complexity. As expected, the readily oxidizable substrate acetate produced three times more power compared to the more complex substrates OECD media and WW. This is consistent with the findings of Liu et al. (2005) who reported 66% higher power generation in MFCs fed acetate than MFCs fed butyrate, and numerous other studies that have shown acetate-fed MFC to have higher performance than MFC fed propionate, glucose, lactate or synthetic wastewater (Jung and Regan, 2007; Chae et al., 2009; Liu et al., 2009; Ebadinezhad et al., 2019). In comparison with OECD media, WW fed reactors showed a slightly higher performance both with and without pre-enrichment on acetate (Figures 1–3). Single step enrichment on acetate and two step enrichment on acetate (a non-fermentable substrate) followed by glucose (a fermentable substrate), or enrichment on a mixture of acetate or glucose did not lead to enhanced MFC performance and a decrease in voltage was observed when changing from acetate to a more complex substrate (Zhang et al., 2011; Wang et al., 2012; Park et al., 2017). By contrast Park et al. (2017) reported higher current production from wastewater when reactors were previously sequentially enriched with acetate and glucose.

The impact of acetate pre-enrichment on COD removal was also affected by the nature of the substrate. Enrichment on acetate had a larger impact on COD removal in WW-fed reactors compared to OECD-fed reactors (Figures 3A,B) This may be a consequence of differences in composition of the two different substrates. While broadly similar in bulk composition (Supplementary Figure S8) the COD in OECD synthetic wastewater has a high content of peptone. This likely explains the high levels of *Cetobacterium* detected in OECD-fed and acetate-enriched OECD MFC compared to MFC fed real wastewater (Figure 5). *Cetobacterium* ferments peptone with acetate as a major end product (Finegold et al., 2003; Edwards et al., 2015; Wang et al., 2021) and as a consequence OECD-fed MFC may in effect already be pre-enriched on acetate. One might therefore expect to see less difference in COD removal between acetate-enriched, OECD wastewater fed MFC and MFC fed solely OECD wastewater compared to acetate-enriched, real wastewater fed MFC and MFC fed solely real wastewater (Figures 3A,B). Liu et al. (2009) detected comparable COD removal efficiencies for batch acetate-fed and OECD-fed reactors but consistent with the results reported here, Park et al. (2017) observed a lower COD removal in wastewater-fed reactors (68% average COD-removal) compared to acetate (77% average COD-removal) (Figure 1).

OECD synthetic wastewater is similar to domestic wastewater in broad composition (Supplementary Figure S7). Though as might be expected real wastewater is more variable than the OECD medium and some anion and formic acid concentrations differ between the two substrates. Though given that OECD wastewater comprises primarily peptone and yeast extract its specific composition will likely have less polymeric carbohydrate and fermentable sugars than real wastewater. The higher voltage produced in WW fed reactors could be due therefore to the wastewater including a higher proportion of substrates that can be readily hydrolysed and fermented to produce acetate. It may also be already partially hydrolysed and therefore more readily degradable than the protein rich synthetic OECD medium, and thus provide a better substrate for exoelectrogens at the anode which predominantly oxidize simple organic compounds such as acetate (Liu et al., 2009).

The substrate fed to MFCs, and pre-enrichment on acetate had a distinct influence on coulombic efficiency. Acetate-fed reactors had the highest CE with CE for WW- and OECD-fed reactors being similar, but lower than acetate-fed reactors. However, pre-enrichment with acetate more than doubled the CE in WW-fed reactors and increased CE by *ca.* 6% in OECD-fed reactors (Figure 3).

### Effect of substrate, inoculum and acetate pre-enrichment on the anodic microbial community

The diversity of microbial communities in MFC reactors was considerably lower than in control reactors (Supplementary Figure S5). This reflects selection for exoelectrogens in the anode community compared to biofilms on carbon electrodes in non-polarized reactors operated at open circuit. The diversity of the anodic communities was also lower in MFCs fed lower complexity substrates (community diversity was lowest to highest in the following order; Acetate-fed reactors → acetate- enriched OECD-fed reactors → OECD-fed reactors → acetate-enriched WW-fed reactors → WW-fed reactors). This is consistent with the greater diversity of compounds present in the more complex substrates leading to greater opportunity for a wider range of organisms that utilize different compounds. Similar trends have been observed previously (Park et al., 2017). High diversity of microbial communities in activated sludge and River Tyne sediment inocula (Supplementary Figure S5) show that both are a rich reservoir of microbial taxa for the development of anodic biofilms in the reactors. The MFC effluent inoculum on the other hand is already enriched for exoelectrogens but interestingly, irrespective of the inoculum used the performance of MFC fed a particular substrate was very similar (Figures 1–3).

Clustering of microbial communities at Family-level based on the 16S rRNA genes recovered from anodic biofilms, using Bray Curtis similarity, shows clear evidence of differences in microbial community structure between the three different inocula used, anodic communities from MFC fed different substrates and corresponding communities enriched on carbon electrodes from control reactors enriched and operated under open circuit conditions (Figure 4). Communities from electrodes from open-circuit control reactors cluster together based on substrate as do the MFC reactors (Figure 4). Velasquez-Orta and colleagues observed similar patterns where anodic communities from MFCs fed acetate, glucose or starch clustered by feed substrate (Velasquez-Orta et al., 2011).

Despite the different inocula and substrates used, bacteria from the genus *Geobacter* were the single most abundant microorganisms enriched in anodic biofilms. This was not surprising as abundant evidence shows that wastewater fed MFC reactors frequently enrich *Geobacter* spp. in their anodic biofilms (Zhu et al., 2014a; Du et al., 2018; Heidrich et al., 2018; Tian et al., 2022). Recently Du et al. (2018) confirmed that the intensity of the electric field around an electrode rather than the anode potential selects for exoelectrogens in anodic biofilms (Zhu et al., 2014a). Du et al. (2018) observed up to 76% relative abundance of *Geobacter* spp. in an electrode biofilm, comparable to the results reported in the present study (*ca.* 70% *Geobacter* spp. in acetate-fed MFC reactors) and *Geobacter* spp. were slightly enriched in control reactors fed acetate (7 ± 0.36% *Geobacter* spp.) and control reactors enriched on acetate and switched to OECD (2.9 ± 1.29% *Geobacter* spp.) or WW (1.9 ± 0.74% *Geobacter* spp.) but essentially absent on non-polarized electrodes fed OECD or WW with 0.65 ± 0.34% and 0.31 ± 0.12% *Geobacter* spp. respectively.

Other taxa, enriched above 1% relative abundance, varied between reactors using different substrates (Figure 5). A *Rhodopseudomonas* sp., was enriched (*ca.* 10% relative abundance; Figure 5) in the acetate, acetate-enriched OECD-fed and OECD-fed reactors which were inoculated with MFC effluent, is a close relative to *Rhodopseudomonas palustris* which is abundant in many ecosystems. This bacterium is metabolically very versatile and can switch between different forms of metabolism (photoautotrophy, photoheterotrophy, chemoautotrophy, and chemoheterotrophy) depending on environmental conditions. It is also the focus of extensive research for it’s potential to break down aromatic compounds and waste in polluted environments and production of hydrogen as a product of nitrogen fixation (Mehrabi et al., 2001; Larimer et al., 2004). *Rhodopseudomonas palustris* DX-1 has been isolated from an acetate-fed MFC inoculated with wastewater. Strain DX-1 was tested for its ability to generate electricity in a MFC and delivered high power density with a pure culture giving slightly higher power (1,170 ± 30 mW/m^2^) than a mixed microbial community enriched from the same wastewater inoculum (1,100 ± 40 mW/m^2^) (Xing et al., 2008). Strain DX-1 was shown to generate high power densities with acetate as an electron donor along with a range of other low molecular weight organic acids and alcohols also being used for electricity generation (Xing et al., 2008). It was also demonstrated that strain DX-1 can generate electricity with yeast extract as a substrate (Xing et al., 2008). This is of particular note given that in this study the *Rhodopseudomonas* sp. was only appreciably enriched in MFC effluent-inoculated MFC fed acetate or OECD synthetic wastewater which contains high levels of yeast extract. It is therefore conceivable that the MFCs studied where the *Rhodopseudomonas* sp. was present at high relative abundance, that it contributed to electricity generation.

Although the synthetic OECD wastewater medium is comparable in broad composition to the domestic wastewater used in this study (Supplementary Figure S8), both real WW and OECD synthetic wastewater enriched different microbial communities in the anodic biofilms. Bacteria from the genus *Cetobacterium* were enriched in OECD-fed and acetate-enriched OECD-fed reactors. Bacteria from the genus *Cetobacterium* ferment peptone and carbohydrates and produce acetic acid and, in smaller amounts, propionic, butyric, lactic, and succinic acid as a fermentation end products (Edwards et al., 2015; Wang et al., 2021). One of the main components of OECD medium is peptone, and enrichment of *Cetobacterium* spp. related to *Cetobacterium somerae*, a novel species isolated from human feces which produced 75% acetic acid, 18% propionic and some butyric acid in peptone-yeast broth (Finegold et al., 2003). *Cetobacterium* clearly forms a component of an anaerobic food chain, generating acetate from peptones in OECD medium which is then utilized by electrogenic *Geobacter* spp. Members of the Family *Rikenellaceae,* have been isolated from fecal samples and the digestive tract of a wide range of animals (Graf, 2014). Known species of the family *Rikenellaceae* are fermenters and produce acetic acid, propionic acid, succinic acid and alcohols from glucose, lactose, mannose and melibiose (Graf, 2014). *Rikenellaceae* are present at elevated relative abundance in reactors fed complex substrates (those containing WW or OECD synthetic wastewater) and was most abundant (~ 2% relative abundance) in reactors fed acetate-enriched OECD or OECD medium. By contrast it was present at much lower relative abundance in MFC fed acetate alone (Figure 5) further suggesting that it may have a role in converting complex organic compounds to fermentation products that promote electrogenesis.

Bacteria from the Family *Desulfobulbaceae,* were enriched to the greatest extent in WW and acetate-enriched WW-fed reactors. Members of *Desulfobulbaceae* are strict anaerobes and are capable of both respiratory and fermentative metabolism oxidizing simple organic compounds like lactate, pyruvate, propionate, and alcohols incompletely to acetate (Samain et al., 1984; Lien et al., 1998; Sorokin et al., 2008; Kuever, 2014) and therefore may play a role in generating acetate that is utilized by electrogens like *Geobacter* spp. Given their ability to partially oxidize other volatile fatty acetates and alcohols to acetate, they may have a role as secondary fermenters of acids and alcohols produced by other members of the anodic microbial communities fed wastewater such as *Romboustia* (see below). Some filamentous bacteria from the *Desulfobulbaceae* are known as cable bacteria and have also been shown to conduct electrons over centimeter long distances by electrically coupling sulfide oxidation and oxygen reduction in marine sediments (Pfeffer et al., 2012; Reguera, 2012; Marzocchi et al., 2014; Larsen et al., 2015; Trojan et al., 2016; Burdorf et al., 2017) and may potentially have a role as electrogens in the MFC where they are found. Bacteria from the genus *Romboutsia,* like members of the *Desulfobulbaceae*, were mainly enriched in wastewater-fed reactors. *Romboustia* has previously been identified in the intestine of mammalian hosts. *Romboutsia* strains can ferment carbohydrates to acetate, formate, lactate and hydrogen via glycolysis, and possess the non-oxidative pentose phosphate pathway (Gerritsen et al., 2014, 2019). These properties would be consistent with a role in providing electron donors for electrogens in environments where complex substrates must be converted to acteate to support electrogens in anodic communities and potentially generating substrates such as lactate that can be utilized by members of the *Desulfobulbaceae* which can convert lactate to acetate. The anodic community of WW-fed reactors was also enriched in uncultured bacteria from the order *Bacteroidales* which consist of anaerobes commonly found in the human gut (Krieg et al., 2010). Members of the order Bacteroidales are by far the most studied species within the phylum Bacteroidetes *Bacteroides thetaiotaomicron* which is commonly found in the human gut flora, can digest complex carbohydrates. It is considered as the type species for investigating polysaccharide degradation within the family Bacteroidales (Porter et al., 2018; Tan et al., 2018). As a commensal bacterium, *B. thetaiotaomicron* produces short-chain carbohydrates and organic acids which can be absorbed by the host as a source of energy (Porter et al., 2018) or further used for electron production in an anodic biofilm.

Based on these observations it is apparent that the taxa enriched in the MFC reactors either were directly involved in electricity generation as exoelectrogenic bacteria (*Geobacter* spp. and putatively members of the family *Desulfobulbaceae* and *Rhodopseudomonas* spp.) or comprised a syntrophic microbial community providing electron donors for the electrogens in the anodic biofilm.

## Conclusion

We have systematically evaluated the concept that MFC performance can be improved by pre-enriching the anode community with electrogens such as *Geobacter* by feeding with acetate prior to more complex substrates. We demonstrated that this approach does lead to a higher proportion of *Geobacter* spp. in the anodic biofilm. However, the increased levels of *Geobacter* did not lead to enhancement of MFC performance except that acetate pre-enriched MFCs subsequently fed complex substrates did exhibit greater coulombic efficiency than MFC fed with complex substrates from the start. The inoculum used also had little effect on the final performance of the MFC even though MFCs inoculated with MFC effluent did have a slightly higher level of *Geobacter* in their anodic biofilms. Efforts to improve the performance of anodic microbial communities in MFCs utilizing complex organic substrates should therefore focus on enhancing the activity of organisms driving hydrolysis and fermentation rather than directly targeting the terminal oxidizing electrogens.

## Data availability statement

The original contributions presented in the study are included in the article/supplementary materials, further inquiries can be directed to the corresponding author.

## Author contributions

BC and IH conceived and designed the study. EY, TC, KS, and IH contributed to funding acquisition and supervision. BC, EM, PI, MS, and CM contributed to the investigation and methodology. BC analyzed the data. BC and IH wrote the manuscript. All authors contributed to the article and approved the submitted version.
