# Supplementary material for: Does pre-enrichment of anodes with acetate to select for Geobacter spp. enhance performance of microbial fuel cells when switched to more complex substrates?
Source: Front Microbiol. 2023 Nov 23;14:1199286. doi: 10.3389/fmicb.2023.1199286 (PMC10702221; doi:10.3389/fmicb.2023.1199286)
Supplement: Supplementary file 1 [file Data_Sheet_1.docx]

**Supplementary Information**

Figure S1: Internal resistance of the control reactors. Control reactors were single MFC reactors set up for every condition but under open circuit potential. The reactors were fed acetate medium (Acetate), synthetic wastewater medium (OECD), or municipal wastewater (WW) and inoculated using MFC effluent (MFC), Tyne sediment (TS) or activated sludge (AS). Additionally, six control reactors were enriched on acetate and then three each were switched to OECD or WW respectively.

Figure S2: Open circuit potential of the control reactors averaged over the batches. Control reactors were single MFC reactors set up for every condition but under open circuit potential. The reactors were fed acetate medium (Acetate), synthetic wastewater medium (OECD), or municipal wastewater (WW) and inoculated using MFC effluent (MFC), Tyne sediment (TS) or activated sludge (AS). Additionally, six control reactors were enriched on acetate and then three each were switched to OECD or WW respectively.

Figure S3: Percentage COD removal of the control reactors over the batches. Control reactors were single MFC reactors set up for every condition but under open circuit potential. The reactors were fed acetate medium (Acetate), synthetic wastewater medium (OECD), or municipal wastewater (WW) and inoculated using MFC effluent (MFC), Tyne sediment (TS) or activated sludge (AS). Additionally, six control reactors were enriched on acetate and then three each were switched to OECD or WW respectively. For these six the percentage COD removal was measured twice once while enriching on acetate (blue) and a second time after switching to OECD and WW as substrate respectively (orange).

Figure S4: Current flow at OCP for the control reactors. Control reactors were single MFC reactors set up for every condition but under open circuit potential. The reactors were fed acetate medium (Acetate), synthetic wastewater medium (OECD), or municipal wastewater (WW) and inoculated using MFC effluent (MFC), Tyne sediment (TS) or activated sludge (AS). Additionally, six control reactors were enriched on acetate and then three each were switched to OECD or WW respectively. *The data logger dataTaker DT85 from OMNI Instruments is a multiplexer which means a current is only applied to the reactor while a measurement takes place. The data logger has an internal resistance (internal impedance) of >100MΩ.


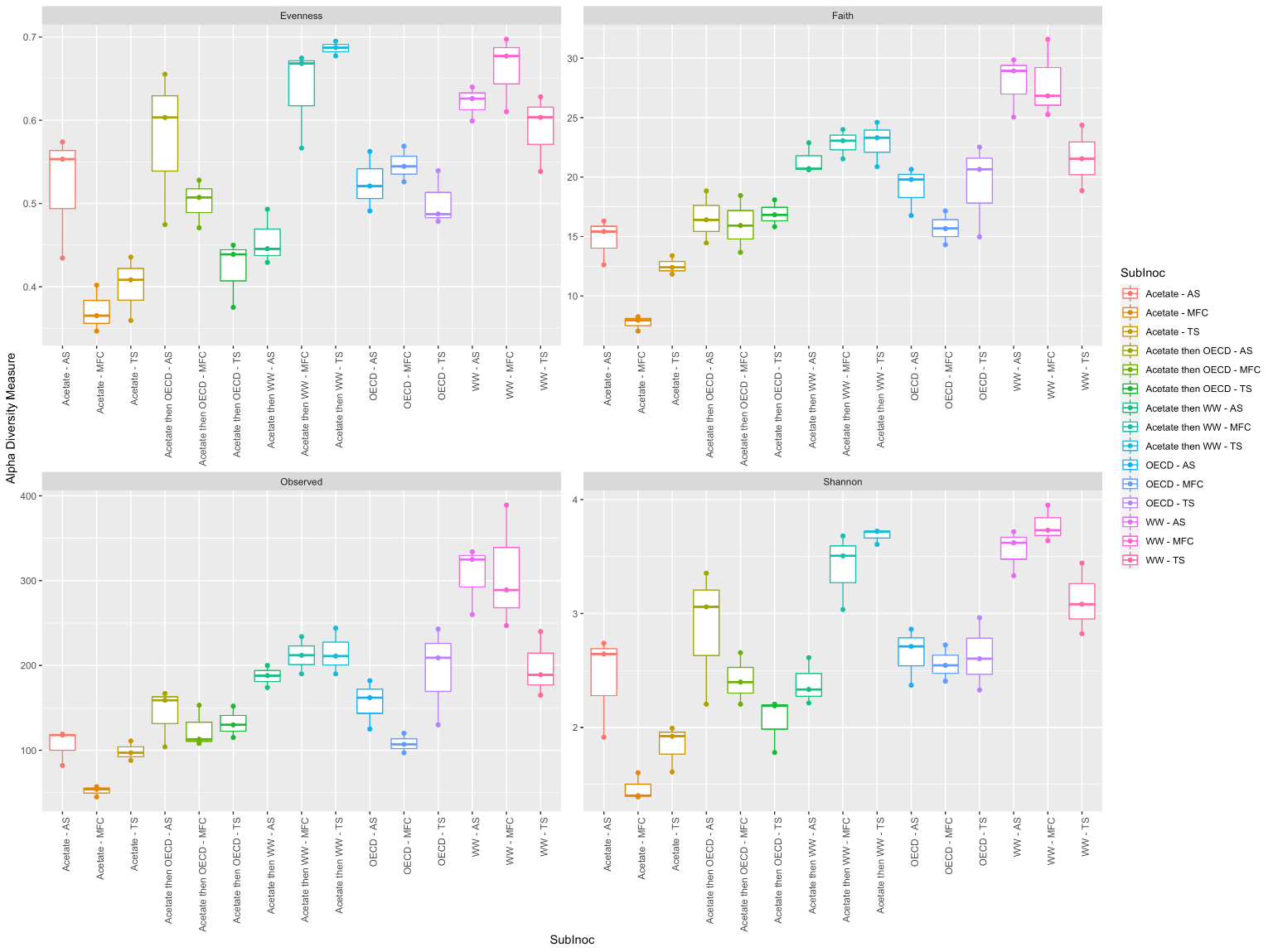
**
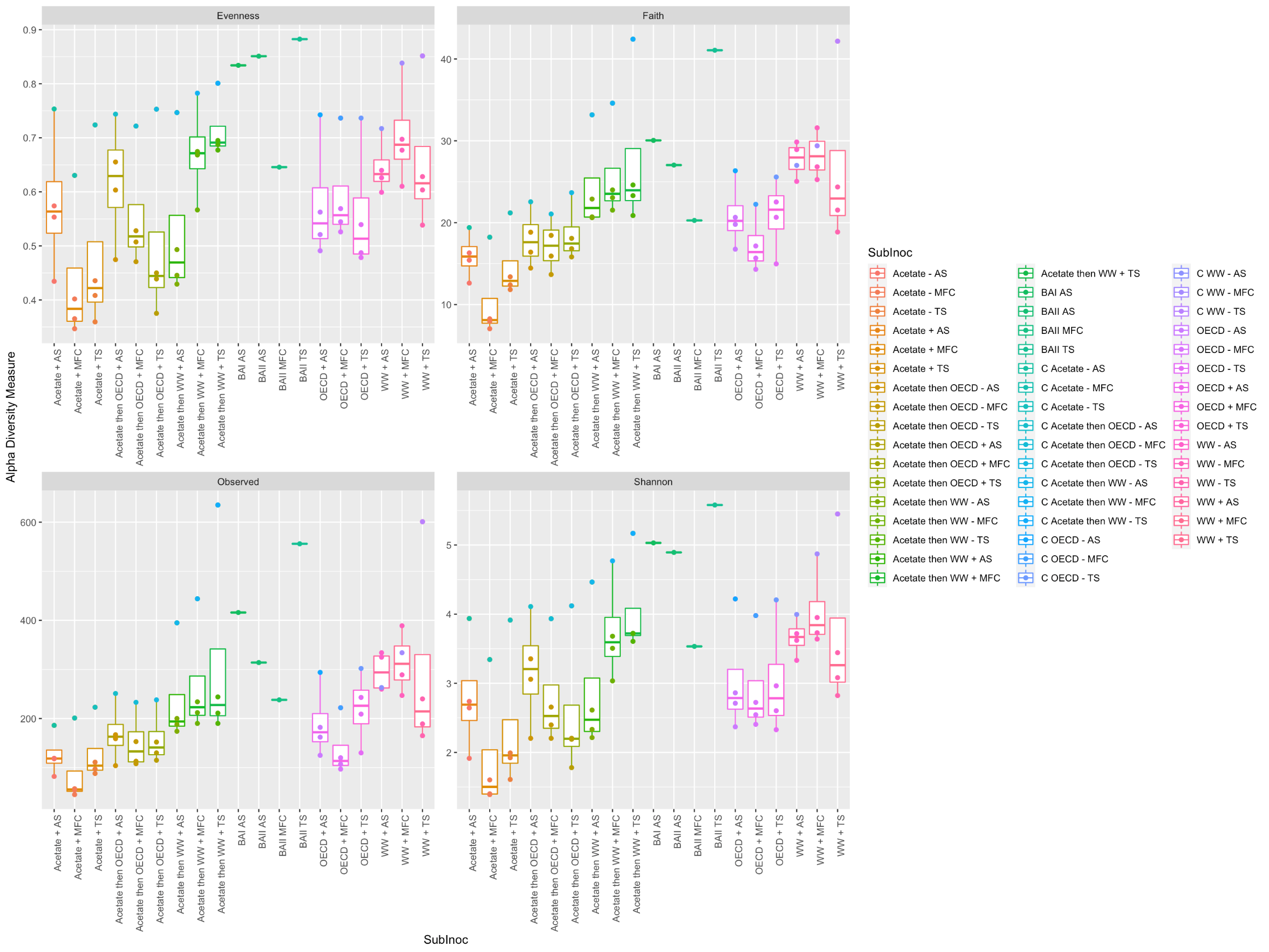

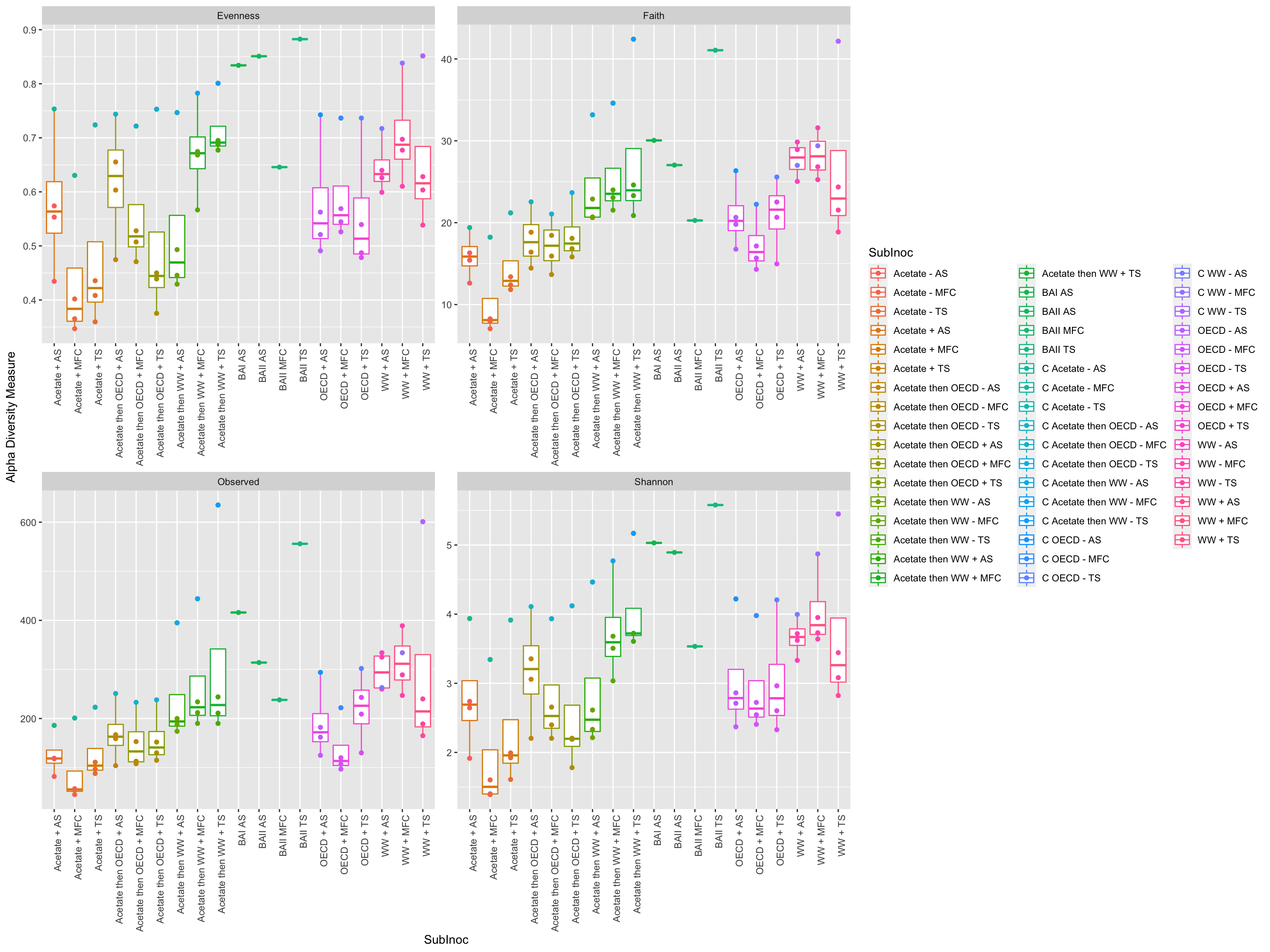
**

**Figure S5:** Shannon Index as alpha diversity measure of the reactors. The triplicate MFC reactors for the different conditions can be seen as box plots. The single control reactors for each condition are the outlier (single dot above showing higher alpha diversity) above the boxplot of the same conditions. The inoculum samples are BAI AS, BAII AS, BAII MFC and BAII TS.


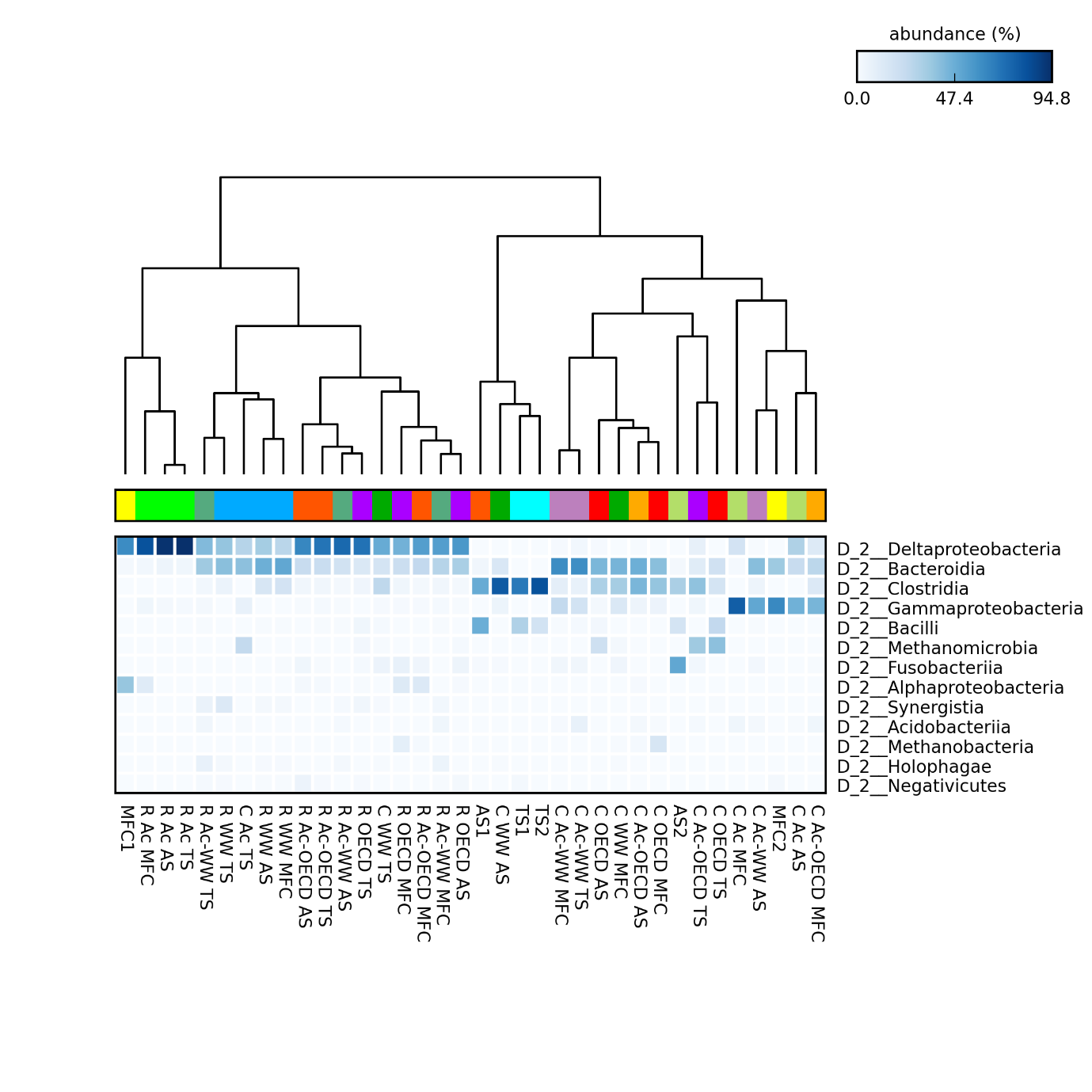


Figure S6: Dendogram and heatmap of class of bacteria enriched in the reactors and controls and seen in the inocula. The triplicate MFC reactors (R stands for MFC reactor) under a 100Ω resistor were fed acetate medium (Acetate), synthetic wastewater medium (OECD), or municipal wastewater (WW) and inoculated using MFC effluent (MFC eff), Tyne sediment (TS) or activated sludge (AS). Additionally, six reactors were enriched on acetate and then three each were switched to OECD (Ac-OECD) or WW (Ac-WW) respectively. Control reactors were single MFC reactors set up for every condition but under open circuit potential. The control reactors have a C in front of the description. Inocula consisted of MFC effluent (MFC1 and MFC2), activated sludge (AS1 and AS2) and Tyne sediment (TS1 and TS2).

Figure S7: Comparison of municipal wastewater (WW) and synthetic OECD wastewater medium. The measured concentrations showed that the synthetic wastewater used is comparable to domestic wastewater.
